# Das kombinierte Modell der Entscheidungsassistenz: Ein Mittel zur ethisch vertretbaren Umsetzung von Artikel 12 der UN-Behindertenrechtskonvention in der Psychiatrie

**DOI:** 10.1007/s00115-022-01384-1

**Published:** 2022-09-19

**Authors:** Matthé Scholten, Jakov Gather, Jochen Vollmann

**Affiliations:** 1 Institut für Medizinische Ethik und Geschichte der Medizin, Ruhr-Universität Bochum, Markstr. 258a, 44799 Bochum, Deutschland; 2 Klinik für Psychiatrie, Psychotherapie und Präventivmedizin, LWL-Universitätsklinikum, Ruhr-Universität Bochum, Bochum, Deutschland

**Keywords:** Menschenrechte, Gleichbehandlung, Einwilligungsfähigkeit, Stellvertretende Entscheidungsfindung, Zwang, Human rights, Equality, Mental capacity, Proxy decision making, Coercion

## Abstract

**Hintergrund:**

Die Auslegung von Artikel 12 der Behindertenrechtskonvention der Vereinten Nationen (Art. 12 UN-BRK) durch den Ausschuss für die Rechte von Menschen mit Behinderungen hat zu einer Kontroverse über die Umsetzung des Artikels in der Psychiatrie geführt.

**Fragestellung:**

Wie kann Art. 12 UN-BRK auf ethisch vertretbare Weise in der Psychiatrie umgesetzt werden?

**Material und Methode:**

Es wurde eine empirisch und rechtlich informierte konzeptionelle und ethische Analyse durchgeführt.

**Ergebnisse:**

Das vorgeschlagene kombinierte Modell der Entscheidungsassistenz gewährleistet die Anerkennung von Menschen mit psychischen Erkrankungen als Rechtssubjekt, deren Gleichbehandlung im Hinblick auf die Erteilung einer Einwilligung sowie die Bereitstellung von Entscheidungsassistenz. Nach diesem Modell dürfen Zwangsbehandlungen nur unter Achtung des Willens und der Präferenzen der Person und weiteren Voraussetzungen der Verhältnismäßigkeit und unabhängiger Überprüfung durchgeführt werden.

**Schlussfolgerungen:**

Art. 12 UN-BRK kann nach dem kombinierten Modell der Entscheidungsassistenz auf ethisch vertretbare Weise in der Psychiatrie umgesetzt werden.

## Abstract (en)

**Background:**

The interpretation of Article 12 of the United Nations (UN) Convention on the Rights of Persons with Disabilities (CRPD) by the UN Committee on the Rights of Persons with Disabilities led to a controversy over the implementation of the article in psychiatry.

**Objective:**

How can Article 12 CRPD be implemented in psychiatry in an ethically justifiable way?

**Material and method:**

An empirically and legally informed conceptual and ethical analysis was carried out.

**Results:**

The suggested combined supported decision making model ensures the recognition of people with mental disorders as persons before the law, their equal treatment in the informed consent process and the provision of supported decision making. According to this model, coercive treatment can only be carried out in accordance with the will and preferences of the person and is subject to further conditions of proportionality and review by an independent body.

**Conclusion:**

The combined supported decision making model makes an ethically justifiable implementation of Article 12 CRPD possible in psychiatry.

Die Auslegung von Artikel 12 der Behindertenrechtskonvention der Vereinten Nationen (Art. 12 UN-BRK) durch den Ausschuss für die Rechte von Menschen mit Behinderungen hat zu einer Kontroverse über die Umsetzung des Artikels in der Psychiatrie geführt. In diesem Beitrag wird dafür argumentiert, dass Art. 12 UN-BRK mithilfe des kombinierten Modells der Entscheidungsassistenz auf ethisch vertretbare Weise in der Psychiatrie umgesetzt werden kann.

## Hintergrund

Die UN-BRK wurde 2006 von den Vereinten Nationen verabschiedet und ist 2008 in Kraft getreten. Deutschland hat die UN-BRK 2009 vorbehaltslos ratifiziert [1]. Die UN-BRK zielt darauf ab, „den vollen und gleichberechtigten Genuss aller Menschenrechte und Grundfreiheiten durch alle Menschen mit Behinderungen zu fördern, zu schützen und zu gewährleisten“ (Art. 1 UN-BRK). Unter „Menschen mit Behinderungen“ fallen auch Menschen mit kognitiven Einschränkungen oder psychischen Erkrankungen (Art. 1 UN-BRK). Damit hat die UN-BRK eine sehr hohe Relevanz für die Psychiatrie.

Es gibt eine große Kontroverse über die Umsetzung von Art. 12 UN-BRK in der Psychiatrie

Die erste allgemeine Bemerkung des Ausschusses für die Rechte von Menschen mit Behinderungen (im Folgenden: der Ausschuss) hat zu einer großen Kontroverse über die Umsetzung von Art. 12 UN-BRK in der Psychiatrie geführt [2–6]. In der Bemerkung hat der Ausschuss Art. 12 UN-BRK dahingehend ausgelegt, dass das Konzept der Einwilligungsfähigkeit hinfällig sei und die Praxis der stellvertretenden Entscheidungsfindung, der Zwangsbehandlung und der Anwendung freiheitsentziehender Maßnahmen (wie z. B. Unterbringung, Fixierung und Isolierung) abgeschafft werden müsse [7].

In diesem Beitrag wird zuerst die Kontroverse um Art. 12 UN-BRK dargestellt. Danach soll gezeigt werden, wie Art. 12 UN-BRK auf ethisch vertretbare Weise in der Psychiatrie umgesetzt werden kann. Als Mittel zur Umsetzung schlagen wir das von uns an anderer Stelle vorgestellte „kombinierte Modell der Entscheidungsassistenz“ („combined supported decision-making model“) vor [3, 8–10]. Abschließend leiten wir einige klinische Empfehlungen aus dem Modell ab.

## Die Kontroverse um Art. 12 UN-BRK

Im Folgenden wird die Bedeutung von Art. 12 UN-BRK sowie der ersten allgemeinen Bemerkung des Ausschusses für die Psychiatrie hervorgehoben.

### Art. 12 UN-BRK

In unserer Darstellung fokussieren wir uns ausschließlich auf die Implikationen des Artikels für die gesundheitliche Versorgung von Menschen mit psychischen Erkrankungen. In Art. 12 Abs. 1 UN-BRK wird die Anerkennung von Menschen mit psychischen Erkrankungen als Rechtssubjekt bekräftigt. Anlass für die Kontroverse über Art. 12 UN-BRK war Abs. 2, welcher besagt, dass Menschen mit psychischen Erkrankungen „in allen Lebensbereichen gleichberechtigt mit anderen Rechts- und Handlungsfähigkeit (‚legal capacity‘) genießen“ (Art. 12 Abs. 2 UN-BRK). Da das Erteilen einer informierten Einwilligung eine Ausübung der Rechts- und Handlungsfähigkeit darstellt, bedeutet dies, dass Menschen mit psychischen Erkrankungen gleichberechtigt mit anderen die Möglichkeit gewährt werden muss, eine informierte Einwilligung zu erteilen oder zu verweigern [3].

Die UN-BRK ist geprägt von einem Bewusstsein dafür, dass Menschen mit Behinderungen durch gesellschaftliche Barrieren oder fehlende Unterstützung nicht immer in der Lage sind, ihre Rechts- und Handlungsfähigkeit auszuüben. Art. 12 Abs. 3 UN-BRK verpflichtet Vertragsstaaten dazu, geeignete Maßnahmen zu treffen, um Menschen mit Behinderungen Zugang zu der dazu notwendigen Unterstützung zu verschaffen. Wie Menschen mit körperlichen Behinderungen mittels barrierefreier Gebäudezugänge oder Menschen mit Sinnesbehinderungen mittels Blindenschrift oder Gebärdensprache bei der Ausübung ihrer Rechts- und Handlungsfähigkeit unterstützt werden können, so können Menschen mit kognitiven Einschränkungen oder psychischen Erkrankungen mittels Maßnahmen der Entscheidungsassistenz in die Lage versetzt werden, selbstbestimmte Entscheidungen zu treffen.

### Die Auslegung von Art. 12 UN-BRK durch den Ausschuss

Mit Inkrafttreten der UN-BRK wurde gemäß Art. 34 UN-BRK der eingangs erwähnte Ausschuss für die Rechte von Menschen mit Behinderungen eingesetzt. Vertragsstaaten sind zur regelmäßigen Berichterstattung über ihre Maßnahmen zur Umsetzung der UN-BRK an den Ausschuss verpflichtet (Art. 35 UN-BRK). Der Ausschuss ist befugt, sowohl Empfehlungen an einzelne Vertragsstaaten (Art. 36 Abs. 1 UN-BRK) als auch allgemeine Empfehlungen zur Umsetzung der UN-BRK abzugeben (Art. 39 UN-BRK).

In seiner ersten allgemeinen Bemerkung zu Art. 12 UN-BRK lehnt der Ausschuss das Konzept der Einwilligungsfähigkeit und die damit einhergehende Unterscheidung zwischen einwilligungsfähig und einwilligungsunfähig als diskriminierend, unwissenschaftlich und nicht objektiv ab [7]. In der Bemerkung heißt es, dass „alle Menschen (unabhängig von Behinderung oder Entscheidungsfähigkeit) eine inhärente rechtliche Handlungsfähigkeit haben“ [7] und daher jederzeit selbst die Möglichkeit haben müssen, ihre Einwilligung zu erteilen oder eine Behandlung abzulehnen.

In psychischen Krisensituationen (z. B. während einer psychotischen, manischen oder depressiven Episode) treffen Menschen manchmal Entscheidungen, die sie nicht getroffen hätten, wenn sie in der Lage gewesen wären, die möglichen Folgen ihrer Entscheidungen angemessen zu beurteilen. Der Ausschuss hält jedoch an seiner radikalen Position fest: „Die individuelle Autonomie und die Fähigkeit von Menschen mit Behinderungen, Entscheidungen zu treffen“, so heißt es in der Bemerkung, „müssen jederzeit, auch in Krisensituationen, geachtet werden“, denn auch Menschen mit psychischen Erkrankungen haben „[das Recht], Risiken einzugehen und Fehler zu machen“ [7]. Zur Praxis der stellvertretenden Entscheidungsfindung heißt es unmissverständlich, dass „Vertragsstaaten dazu verpflichtet [sind], stellvertretende Entscheidungen im Namen von Menschen mit Behinderungen zu untersagen“ [7]. Eine solche Praxis müsse dem Ausschuss zufolge vollständig vom Paradigma der Entscheidungsassistenz abgelöst werden [7].

### Kritik an der Auslegung des Ausschusses

Die dargelegte Auslegung von Art. 12 UN-BRK durch den Ausschuss hat starke Kritik ausgelöst [2–6]. Von Kritikern, die die UN-BRK und ihre grundlegenden Ziele befürworten, wurde der Einwand erhoben, dass die durch den Ausschuss vorgelegte Auslegung des Art. 12 UN-BRK den grundlegenden Zielen des Übereinkommens zuwiderlaufe.

Dieser Einwand lässt sich gut am Beispiel psychiatrischer Patientenverfügungen erläutern [10]. Nehmen wir an, dass eine Person mit einer psychischen Erkrankung nach einer stationären Behandlung in einem psychiatrischen Krankenhaus eine Patientenverfügung verfasst, in der sie festlegt, dass sie aufgrund erlebter starker Nebenwirkungen in zukünftigen Krisensituationen, die mit akuter Eigengefährdung einhergehen, nicht wieder mit Haloperidol, sondern mit Olanzapin behandelt werden will. Stellen wir uns nun vor, dass die Person nach einiger Zeit wieder psychotisch exazerbiert, wegen akuter Eigengefährdung in einem psychiatrischen Krankenhaus aufgenommen wird und dort aufgrund eines Vergiftungswahns jegliche medikamentöse Behandlung ablehnt.

Nach der vom Ausschuss vorgesehenen Umsetzung der UN-BRK müssten psychiatrische Professionelle in diesem Fall den aktuell geäußerten Wunsch, keine medikamentöse Behandlung zu bekommen, befolgen. Dies würde die Wirksamkeit der von der Person vor dem Hintergrund ihrer eigenen persönlichen Erfahrungen und Wertvorstellungen selbstbestimmt in der Patientenverfügung festgelegten Wünsche untergraben [10]. Die Auslegung des Ausschusses begrenzt daher die Möglichkeit von Menschen mit psychischen Erkrankungen, ein selbstbestimmtes Leben zu führen, und kann zu erheblichen gesundheitlichen und psychosozialen Schäden für sie führen [3]. Im Hinblick auf diese möglichen gesundheitlichen Schäden kann überdies argumentiert werden, dass die vom Ausschuss vorgesehene Umsetzung der UN-BRK in bestimmten Fällen Menschen mit psychischen Erkrankungen das von der UN-BRK garantierte Recht auf Gesundheit (Art. 25 UN-BRK) vorenthält.

### Rechtliche Einordnung der UN-BRK und der allgemeinen Bemerkung

Mit der Ratifizierung der UN-BRK durch die Bundesrepublik Deutschland ist diese in Deutschland zum geltenden Recht geworden und nimmt den Rang eines einfachen Bundesgesetzes ein [11]. Obwohl die UN-BRK damit nicht über den Regelungen des Betreuungsrechts steht, muss der Gesetzgeber die bestehenden Gesetze auf Vereinbarkeit mit der UN-BRK überprüfen und sie ggf. anpassen. Auch bei der Auslegung der Regelungen des Betreuungsrechts muss die UN-BRK herangezogen werden [11].

Das Bundesverfassungsgericht hat die Bedeutung der Auslegung des Ausschusses rechtlich eingeordnet. Wenngleich solchen Auslegungen ein „erhebliches Gewicht“ zukomme, so das Bundesverfassungsgericht, seien sie „für internationale und nationale Gerichte nicht völkerrechtlich verbindlich“ (BVerfGE 142, 313 [346 Rn. 90]). Zuständig für die Auslegung der UN-BRK sind demnach die nationalen Gerichte. Das Bundesverfassungsgericht hat „dem Art. 12 BRK kein grundsätzliches Verbot für Maßnahmen entnommen, die gegen den natürlichen Willen Behinderter vorgenommen werden und an eine krankheitsbedingt eingeschränkte Selbstbestimmungsfähigkeit anknüpfen“ (BVerfGE 142, 313 [345 Rn. 88]).

## Das kombinierte Modell der Entscheidungsassistenz

Das von uns bereits an anderer Stelle vorgestellte kombinierte Modell der Entscheidungsassistenz [3, 8–10] baut auf einem etablierten Modell der informierten Einwilligung auf [12–14] und ergänzt dieses in verschiedener Hinsicht. Die wesentlichen Aspekte werden im Folgenden kurz dargestellt.

### Respekt der Selbstbestimmung

Das kombinierte Modell der Entscheidungsassistenz gründet primär auf dem Respekt der Selbstbestimmung.

Selbstbestimmung hat erstens einen *instrumentellen* Wert: Wenn Menschen ausführlich über möglichen Nutzen und Risiken der verschiedenen Behandlungsoptionen aufgeklärt worden sind, können wir im Prinzip annehmen, dass sie selbst besser als andere einschätzen können, welche Behandlungsoption am besten zu ihren eigenen Wertvorstellungen und Präferenzen passt. Da das Wohlergehen einer Person zumindest zum Teil in der Erfüllung der eigenen wohlüberlegten Präferenzen besteht [15], können psychiatrische Professionelle in der Regel davon ausgehen, dass sie das Wohlergehen ihrer Patienten fördern, wenn sie die Behandlungswünsche aufgeklärter Patienten befolgen.

Selbstbestimmung hat zweitens einen *inhärenten* Wert: Sie stellt ein Abwehrrecht von Patienten dar, das psychiatrischen Professionellen die Pflicht auferlegt, selbstbestimmte Behandlungsablehnungen zu respektieren, und zwar auch dann, wenn dies dem Wohl ihrer Patienten zuwiderläuft.

Professionelle haben die Pflicht, selbstbestimmte Behandlungsablehnungen zu respektieren

Vor dem Hintergrund des hohen Werts von Selbstbestimmung haben psychiatrische Professionelle außerdem die positive Pflicht, die Selbstbestimmung ihrer Patienten aktiv mittels angemessener Aufklärung und Entscheidungsassistenz zu fördern.

Diese normative Bedeutung der Selbstbestimmung ist im Konzept der informierten Einwilligung operationalisiert worden. Entsprechend diesem Konzept müssen psychiatrische Professionelle vor der Durchführung einer medizinischen Maßnahme die Einwilligung des Patienten einholen. Eine Einwilligung des Patienten ist nur dann gültig, wenn der Patient zuvor über alle für die Therapieentscheidung wesentlichen Informationen aufgeklärt worden ist, er einwilligungsfähig ist und freiwillig, d. h. ohne unangemessenen Druck von anderen, entscheiden kann [12, 16]. Diese Voraussetzungen dienen dazu, eine selbstbestimmte Entscheidungsfindung zu ermöglichen bzw. zu gewährleisten.

### Entscheidungsassistenz

Es gilt eine grundsätzliche Annahme der Einwilligungsfähigkeit. Psychiatrische Professionelle haben daher von der Einwilligungsfähigkeit ihrer Patienten auszugehen, es sei denn, es gibt konkrete Anhaltspunkte für die Notwendigkeit einer Überprüfung der Einwilligungsfähigkeit. Verschiedene empirische Studien haben gezeigt, dass bestimmte psychische Zustände, wie z. B. Psychose, Manie oder schwere Depression, Risikofaktoren für Einwilligungsunfähigkeit sind [17]. Das Vorliegen solcher Risikofaktoren ist nach dem kombinierten Modell weder notwendig noch hinreichend für die Bereitstellung von Entscheidungsassistenz. Wenn eine Person aber solche Risikofaktoren zeigt und es zusätzlich konkrete Hinweise auf eine eingeschränkte Einwilligungsfähigkeit gibt, müssen nach dem kombinierten Modell Maßnahmen der Entscheidungsassistenz bereitgestellt werden.

Maßnahmen der Entscheidungsassistenz sollten bereitgestellt werden

Das Konzept der Entscheidungsassistenz umfasst alle Maßnahmen, die angewendet werden können, um Menschen mit eingeschränkter Einwilligungsfähigkeit in die Lage zu versetzen, selbstbestimmte Entscheidungen zu treffen. Die empirische Evidenz für die Effektivität von Maßnahmen der Entscheidungsassistenz bei psychischen Erkrankungen ist zwar noch begrenzt [18], aber in diesem Kontext kann z. B. an verbesserte Einwilligungsverfahren und die Einbeziehung von Genesungsbegleitern oder anderen Assistenzpersonen gedacht werden [19].

Obwohl Maßnahmen der Entscheidungsassistenz aus theoretischer Perspektive erst bei Einwilligungsunfähigkeit notwendig und angebracht sind, ist aus klinischer Perspektive eine strukturierte Beurteilung der Einwilligungsfähigkeit schon vor der Bereitstellung der Entscheidungsassistenz nicht immer machbar. Eine solche Beurteilung vorab ist ratsam, da sie es ermöglicht, Maßnahmen der Entscheidungsassistenz an die spezifischen Defizite und Ressourcen der Person anzupassen und somit deren Effektivität zu erhöhen. Aus ethischer Perspektive muss der Bereitstellung von Entscheidungsassistenz aber nicht zwangsläufig eine strukturierte Beurteilung der Einwilligungsfähigkeit vorausgehen, da Maßnahmen der Entscheidungsassistenz die Entscheidungsfreiheit einer Person nicht einschränken, sondern diese lediglich unterstützen. Es ist daher ethisch vertretbar, sie schon bei konkreten Hinweisen auf Einwilligungsunfähigkeit zur Verfügung zu stellen.

### Beurteilung der Einwilligungsfähigkeit

Erst wenn es nach der Anwendung von Entscheidungsassistenz weiterhin konkrete Hinweise auf Einwilligungsunfähigkeit gibt, sollte nach dem kombinierten Modell die Einwilligungsfähigkeit der Person beurteilt werden. Das Modell adaptiert das von Grisso und Appelbaum entwickelte fähigkeitsbasierte Konzept der Einwilligungsfähigkeit. Nach diesem Konzept kann man nur auf Einwilligungsunfähigkeit schließen, wenn sich bei einer Beurteilung herausstellt, dass die Person nicht in hinreichendem Maße in der Lage ist,

1. die Aufklärungsinformationen zu verstehen,
2. ihre eigene gesundheitliche Situation sowie die Möglichkeiten der Behandlung realistisch einzuschätzen,
3. die möglichen Folgen der verschiedenen Behandlungsoptionen auf Grundlage der eigenen Werthaltungen und Überzeugungen zu bewerten und gegeneinander abzuwägen und
4. eine eindeutige Entscheidung zu treffen und zu kommunizieren [20].

Stichwortartig können diese vier Fähigkeiten als Informationsverständnis („understanding“), Krankheits- und Behandlungseinsicht („appreciation“), Urteilsvermögen („reasoning“) und Treffen und Kommunizieren einer Entscheidung („evidencing a choice“) bezeichnet werden [14, 20].

Eine strukturierte Beurteilung der Einwilligungsfähigkeit, durchgeführt von Beurteilern, die dazu eine kurze Schulung bekommen haben, hat eine sehr hohe Interraterreliabilität [21, 22]. Die Kriterien der Einwilligungsfähigkeit können so auf eine nichtwillkürliche und intersubjektiv nachvollziehbare Weise angewendet werden. Darüber hinaus ist ein fähigkeitsbasiertes Konzept von Einwilligungsfähigkeit nicht diskriminierend, da es unabhängig von statischen Personenmerkmalen und insbesondere Gruppenzugehörigkeiten angewendet werden kann und es Menschen mit psychischen Erkrankungen im Vergleich zu Menschen ohne psychische Erkrankungen nicht nachteilig behandelt [8].

### Stellvertretende Entscheidungsfindung

Wenn die Bereitstellung geeigneter Maßnahmen der Entscheidungsassistenz für eine Person nicht ausreicht, um Einwilligungsfähigkeit zu erreichen, kann eine selbstbestimmte Entscheidungsfindung nicht erfolgen. Das kombinierte Modell sieht an dieser Stelle die stellvertretende Entscheidungsfindung als nächstbeste Alternative vor. Die stellvertretende Entscheidungsfindung bleibt an den Wertvorstellungen und Überzeugungen der Person orientiert [16].

Die stellvertretende Entscheidungsfindung bleibt an den Wertvorstellungen der Person orientiert

Als Stellvertreter fungiert vorzugsweise eine Person, die den Patienten gut kennt und nicht mit ihm in einem Interessenkonflikt steht. Dies kann ein Angehöriger oder ein guter Freund sein, der von der betroffenen Person als Bevollmächtigter oder rechtlicher Betreuer bestimmt worden ist, oder ein professioneller rechtlicher Betreuer.

Der Stellvertreter hat sich die Frage zu stellen, wie sich die Person in der vorliegenden Situation entscheiden würde, wenn sie einwilligungsfähig wäre. Für die Antwort auf diese Frage gibt es verschiedene Anknüpfungspunkte. Als erstes ist an dieser Stelle eine möglicherweise vorhandene Patientenverfügung zu nennen. Obwohl Patientenverfügungen in der Psychiatrie noch nicht weitverbreitet sind, geht aus empirischen Studien hervor, dass Menschen mit psychischen Erkrankungen Patientenverfügungen stark befürworten [23] und dass die Inhalte der meisten psychiatrischen Patientenverfügungen mit klinischen Standards vereinbar sind [24].

Patientenverfügungen sind der stellvertretenden Entscheidungsfindung zuzuordnen, weil durch ihre Anwendung die in der Patientenverfügung festgelegten Wünsche gegenüber den aktuellen Wünschen der zu diesem Zeitpunkt einwilligungsunfähigen Person priorisiert werden [10]. Gleichwohl ist nach deutschem Recht der Einbezug eines rechtlichen Vertreters für die Umsetzung einer Patientenverfügung, die gültig ist und auf die aktuelle Lebens- und Behandlungssituation zutrifft, nicht erforderlich [11].

Kehren wir kurz zu dem oben im zweiten Abschnitt beschriebenen Beispiel zurück, um das Vorgehen der stellvertretenden Entscheidungsfindung zu erläutern. In diesem Beispiel sollte die Entscheidung gemäß Patientenverfügung prinzipiell für eine Behandlung mit Olanzapin ausfallen, sofern diese Behandlung auch medizinisch indiziert ist. Ob diese Behandlung auch gegen den Willen der Person durchgeführt werden darf, ist eine weitere Frage, denn für eine Zwangsbehandlung müssen zusätzliche Bedingungen erfüllt sein. Zu diesen Bedingungen äußern wir uns im nächsten Abschnitt.

Oft liegt eine Patientenverfügung nicht vor oder ihr Inhalt ist nicht hinreichend bestimmt. In einer solchen Situation hat sich der Stellvertreter an früher geäußerten Behandlungswünschen zu orientieren und zu eruieren, ob sich die Person zu der vorliegenden Situation früher geäußert hat und welche Behandlung sie sich in der Situation wünschen würde. Oft haben sich Menschen aber nicht zu solchen konkreten Lebens- und Behandlungssituationen geäußert. In solchen Fällen muss der Stellvertreter den mutmaßlichen Willen der Person rekonstruieren. Konkret heißt dies, dass er eruieren muss, welche Werthaltungen und Überzeugungen die Person hat und wie sie sich vor diesem Hintergrund in der vorliegenden Situation am ehesten entscheiden würde.

Ärzte werden nur in Situationen zu Entscheidungsträgern, in denen eine Behandlung nicht zeitlich aufgeschoben werden kann und deswegen weder eine Betreuerbestellung noch eine Genehmigung der Maßnahme durch ein Gericht möglich ist. Aus ethischer Sicht ist beim Fehlen jeglicher Hinweise auf den Willen der Person das Wohl der Person ausschlaggebend. Demnach sollte die Person gemäß dem medizinethischen Standard des besten Interesses bzw. nach medizinischer Indikation behandelt werden [16].

## Zwangsbehandlungen vor dem Hintergrund von „Wille und Präferenzen“

In Diskussionen zur Legitimität von Zwangsbehandlungen bedient man sich oft einer bestimmten Terminologie, um Situationen der stellvertretenden Entscheidungsfindung prägnant zu beschreiben. Mit dem Begriff „selbstbestimmter Wille“ werden Entscheidungen einer aufgeklärten und einwilligungsfähigen Person, die sich freiwillig entscheiden kann, bezeichnet. Der Begriff „natürlicher Wille“ verweist im Gegensatz dazu auf die Wünsche, Entscheidungen und Verhaltensweisen einer aktuell einwilligungsunfähigen Person. Der Begriff „vorausverfügter Wille“ wird verwendet, um auf die in einer Patientenverfügung festgelegten Wünsche zu verweisen. Unter dem Begriff des „mutmaßlichen Willens“ versteht man den Willen, der anhand früherer Äußerungen und grundsätzlicher Wertvorstellungen und Überzeugungen der Person ermittelt wird und Entscheidungen umfasst, die eine einwilligungsunfähige Person in einer konkreten Situation am ehesten treffen würde, wenn sie einwilligungsfähig wäre.

Bei der Behandlung von Menschen mit psychischen Erkrankungen kann es zu einem Konflikt zwischen dem natürlichen Willen einerseits und dem vorausverfügten oder mutmaßlichen Willen andererseits kommen. Menschen mit einer psychischen Erkrankung können beispielsweise im einwilligungsunfähigen Zustand während einer psychischen Krise, z. B. einer Manie mit psychotischen Symptomen, eine stationäre Behandlung in einem psychiatrischen Krankenhaus ablehnen, obwohl sie sich für eine solche Behandlung entschieden hätten, wenn sie in der Lage gewesen wären, die möglichen Folgen der Entscheidung abzuschätzen und auf der Grundlage ihrer eigenen Wertvorstellungen und Überzeugungen zu bewerten.

Obwohl das kombinierte Modell dem vorausverfügten und dem mutmaßlichen Willen gegenüber dem natürlichen Willen den Vorzug gibt, folgt hieraus nicht, dass immer, wenn dieser Konflikt auftritt, eine Zwangsbehandlung in Betracht gezogen werden sollte. Eine Zwangsbehandlung darf nach diesem Modell nämlich nur dann erfolgen, wenn weitere Voraussetzungen erfüllt sind. In Anlehnung an rechtliche Diskussionen zum sog. Verhältnismäßigkeitsgrundsatz bezeichnen wir diese als „Voraussetzungen der Verhältnismäßigkeit“.

Erstens muss die Zwangsbehandlung *geeignet* sein, um den drohenden Schaden zu vermeiden. Eine bloße Unterbringung im psychiatrischen Krankenhaus ohne weitere therapeutische Maßnahmen oder intensivierte Betreuung stellt beispielsweise in der Regel keine geeignete Maßnahme dar, um einen Suizid zu verhindern, da der Suizid auch auf der Station erfolgen könnte.

Zweitens muss die Zwangsbehandlung *notwendig* sein, um den drohenden Schaden abzuwenden. Dies beinhaltet, dass solange mildere Maßnahmen, wie z. B. die Anwendung von Deeskalationsmethoden oder Überzeugungsversuchen, noch nicht ausgeschöpft worden sind, eine Zwangsbehandlung nicht erfolgen darf.

Drittens muss die Zwangsbehandlung im Vergleich zu dem zu verhindernden Schaden *verhältnismäßig* sein. Dies bedeutet konkret, dass das Nutzen-Risiko-Verhältnis der Zwangsbehandlung dem Nutzen-Risiko-Verhältnis des Befolgens des natürlichen Willens deutlich überlegen sein muss, was in der Regel nur bei erheblichen Gefahren der Fall sein dürfte. Bei dieser Abwägung sind nicht nur die medizinischen Risiken (z. B. die Nebenwirkungen der verabreichten Medikamente), sondern auch die möglichen psychischen und sozialen Folgen einer Behandlung gegen den Willen zu berücksichtigen. Empirische Studien weisen darauf hin, dass psychische Folgen von Zwangsmaßnahmen oft sehr groß sind [25].

Nur wenn diese drei Voraussetzungen der Verhältnismäßigkeit erfüllt sind, kann nach dem vorgeschlagenen kombinierten Modell eine Zwangsbehandlung ethisch legitimiert werden. Es erscheint plausibel anzunehmen, dass diese weiteren Voraussetzungen nur in seltenen Fällen erfüllt sind. Um eine neutrale Beurteilung der Vertretbarkeit der Durchführung einer Zwangsbehandlung zu gewährleisten und sog. prozedurale Gerechtigkeit zu fördern, ist es aus ethischer Sicht erforderlich, dass eine unabhängige Instanz die Person anhört und beurteilt, ob die Voraussetzungen einer Zwangsbehandlung erfüllt sind.

Art. 12 Abs. 4 UN-BRK besagt, dass gewährleistet werden muss, dass „bei den Maßnahmen betreffend die Ausübung der Rechts- und Handlungsfähigkeit die Rechte, der Wille und die Präferenzen der betreffenden Person geachtet werden“. Im Hinblick auf die oben eingeführte Terminologie stellt sich dabei die folgende konzeptionelle Frage: *Welcher* Wille und *welche* Präferenzen? Der aktuelle Wille, egal ob er von einer einwilligungsfähigen oder einwilligungsunfähigen Person geäußert wird (d. h. egal ob „selbstbestimmt“ oder „natürlich“)? Oder der vorausverfügte bzw. der mutmaßliche Wille?

Der Ausschuss hat sich offenbar auf die erste Antwort auf diese Frage festgelegt: Der aktuelle Wille muss *immer* befolgt werden (vgl. Abschnitt „Die Auslegung von Art. 12 UN-BRK durch den Ausschuss“). Das kombinierte Modell der Entscheidungsassistenz hält hingegen fest, dass der selbstbestimmte Wille immer respektiert und umgesetzt werden muss, sofern eine medizinische Indikation für die gewünschte Behandlung vorliegt. Im Fall eines Konflikts zwischen dem natürlichen und dem vorausverfügten bzw. mutmaßlichen Willen einer aktuell einwilligungsunfähigen Person (a) muss der vorausverfügte bzw. mutmaßliche Wille befolgt werden, sofern im Hinblick auf die dann vorzunehmende Intervention die Voraussetzungen der Verhältnismäßigkeit erfüllt sind; und (b) muss der natürliche Wille befolgt werden, sofern die Voraussetzungen der Verhältnismäßigkeit nicht erfüllt sind. George Szmukler hat dafür argumentiert, dass der hier skizzierte Ansatz im Einklang mit dem von der UN-BRK vorgegebenen Standard von „Wille und Präferenzen“ steht [5, 26].

## Schlussfolgerungen

In diesem Beitrag haben wir gezeigt, dass das von uns vorgeschlagene kombinierte Modell der Entscheidungsassistenz die Anerkennung von Menschen mit psychischen Erkrankungen als Rechtssubjekt, deren Gleichbehandlung im Hinblick auf die Erteilung bzw. Nichterteilung einer Einwilligung und die Bereitstellung von Entscheidungsassistenz gewährleistet. Die Durchführung von Zwangsbehandlungen ist nur in engen Grenzen unter Achtung des Willens und der Präferenzen der Person sowie unter weiteren Voraussetzungen der Verhältnismäßigkeit und unabhängiger Überprüfung ethisch zulässig. Auf diese Weise kann Art. 12 UN-BRK auf ethisch vertretbare Weise in der Psychiatrie umgesetzt werden.

## Fazit für die Praxis

- Psychiatrische Professionelle sollten die Selbstbestimmung von Menschen mit psychischen Erkrankungen mittels einer angemessenen Aufklärung und Bereitstellung von Entscheidungsassistenz aktiv fördern.
- Psychiatrische Professionelle sollten in der Beurteilung der Einwilligungsfähigkeit und der Bereitstellung von Entscheidungsassistenz geschult werden.
- Maßnahmen der Entscheidungsassistenz für Menschen mit psychischen Erkrankungen sollten verstärkt entwickelt und ihre Effektivität empirisch untersucht werden.
