# Erratum zu: Das kombinierte Modell der Entscheidungsassistenz: Ein Mittel zur ethisch vertretbaren Umsetzung von Artikel 12 der UN‑Behindertenrechtskonvention in der Psychiatrie

**DOI:** 10.1007/s00115-022-01401-3

**Published:** 2022-10-25

**Authors:** Matthé Scholten, Jakov Gather, Jochen Vollmann

**Affiliations:** 1 Institut für Medizinische Ethik und Geschichte, der Medizin, Ruhr-Universität Bochum, Markstr. 258a, 44799 Bochum, Deutschland; 2 Klinik für Psychiatrie, Psychotherapie und Präventivmedizin, LWL-Universitätsklinikum, Ruhr-Universität Bochum, Bochum, Deutschland

**Erratum zu:**

**Nervenarzt 2022**

10.1007/s00115-022-01384-1

Die zunächst publizierte Onlineversion des Beitrags enthielt einen fehlerhaften Satz auf S. 6 (linke Spalte, oberer Abschnitt). Bitte beachten Sie die korrigierte Version:

Im Fall eines Konflikts zwischen dem natürlichen und dem vorausverfügten bzw. mutmaßlichen Willen einer aktuell einwilligungs**un**fähigen Person (a) muss der vorausverfügte bzw. mutmaßliche Wille befolgt werden, sofern im Hinblick auf die dann vorzunehmende Intervention die Voraussetzungen der Verhältnismäßigkeit erfüllt sind; und (b) muss der natürliche Wille befolgt werden, sofern die Voraussetzungen der Verhältnismäßigkeit nicht erfüllt sind.

